# Long non-coding RNA MIAT regulates ox-LDL-induced cell proliferation, migration and invasion by miR-641/STIM1 axis in human vascular smooth muscle cells

**DOI:** 10.1186/s12872-021-02048-9

**Published:** 2021-05-20

**Authors:** Gang Ma, Shuting Bi, Pengfei Zhang

**Affiliations:** 1 Deptment of Cardiac Surgury, Zibo Central Hospital, Zibo, 255036 Shandong Peoples Republic of China; 2 Department of Cardiac Surgery, Central Hospital Affiliated to Shandong First Medical University, No.105, Jiefang Road, Jinan, 250013 Shandong Peoples Republic of China; 3 Department of Cardiac Surgery, Jinan Central Hospital Affiliated to Shandong University, Jinan, Shandong Peoples Republic of China

**Keywords:** AS, MIAT, miR-641, STIM1

## Abstract

**Background:**

Atherosclerosis (AS) is a primary cause of coronary heart and vascular diseases. Long non-coding RNAs (lncRNAs) are indicated to regulate AS progression. This study aimed to reveal the biological roles of lncRNA myocardial infarction associated transcript (MIAT) in oxidized low-density lipoprotein (ox-LDL)-induced human vascular smooth muscle cells (VSMCs).

**Methods:**

The RNA levels of MIAT, microRNA-641 (miR-641) and stromal interaction molecule 1 (STIM1) were detected by quantitative real-time polymerase chain reaction (qRT-PCR). The protein levels were determined by western blot analysis. Cell proliferation was assessed by cell colony formation and DNA content quantitation assays. Cell migration and invasion were demonstrated by wound-healing and transwell assays. The putative binding relationships between miR-641 and MIAT or STIM1 were predicted by starbase online database, and identified by dual-luciferase reporter and RNA immunoprecipitation assays.

**Results:**

MIAT and STIM1 expression were substantially upregulated, whereas miR-641 expression was downregulated in ox-LDL-induced VSMCs compared with control groups. Functionally, MIAT silencing attenuated ox-LDL-induced cell proliferation, migration and invasion in VSMCs; however, these effects were impaired by miR-641 inhibitor. STIM1 overexpression also restrained miR-641-mediated impacts on cell proliferation and metastasis under ox-LDL. Mechanistically, MIAT acted as a sponge for miR-641, and miR-641 was associated with STIM1.

**Conclusions:**

MIAT silencing hindered ox-LDL-induced cell proliferation, migration and invasion by downregulating STIM1 expression through binding to miR-641 in VSMCs. The mechanism provided us with a new target for AS therapy.

**Supplementary Information:**

The online version contains supplementary material available at 10.1186/s12872-021-02048-9.

## Background

Atherosclerosis (AS), an inflammatory disease, is a major cause of vascular diseases, including myocardial infarction, ischemic stroke and cardiovascular disease [1]. AS is featured by lipid deposition and contains fat-rich macrophages (m) [2]. Multiple types of cells were revealed to take part in the pathogenesis of AS, such as vascular smooth muscle cells (VSMCs), endothelial cells (ECs) and m [3]. Recent review revealed that SMCs regulated the production of many matrix proteins and promoted the development of the inflammatory response to lipid, suggesting the importance of SMCs in AS progression [4]. Besides, previous research explained that oxidized low-density lipoprotein (ox-LDL) was a vital risk factor for AS [5]. Thus, profoundly revealing the mechanism underlying ox-LDL-induced abnormal transformation of VSMCs can provide us with reliable targets for AS therapy.

Long noncoding RNA (lncRNA) is a non-protein-coding RNA with at least 200 base pairs in size, featured by high conservatism [6]. LncRNA can transfer messages and guide molecule to ribonucleoprotein complexes, thereby playing important roles in biological processes [7]. An increasing number of researches revealed that lncRNAs were involved in the progression of various diseases, including AS [8,10]. LncRNA myocardial infarction associated transcript (MIAT) was also revealed to mediate AS process. As reported, MIAT could inhibit efferocytosis via upregulating clusters of differentiation 47 (CD47) through binding to microRNA-149-5p (miR-149-5p) [11]. MIAT also had ability to modulate diabetes mellitus-caused microvascular dysfunction [12]. However, there were few studies on the development of AS mediated by MIAT [13, 14].

MiRNAs are a category of noncoding RNAs with about 20 nucleotides in size, and regulate gene expression via targeting their noncoding sequences [15]. At present, miRNAs have been unveiled to play crucial parts in modulating cardiovascular cell functions, such as protecting against cardiac dysfunction after cerebral ischemia-reperfusion, inhibiting autophagy, and regulating cardiomyocyte growth [16,18]. Another miRNA, miR-641, only was reported to participate in the regulation of cancer progression. For example, Kong et al. indicated that miR-641 was under-expressed in lung cancer cells, and repressed cell proliferation [19]. In the study of Li and his colleagues, we found the cancer-promoting role of miR-641 in pancreatic cancer by associating with LINC01963 [20]. Stromal interaction molecule 1 (STIM1), an important factor in regulating calcium channels, can dramatically affect intracellular Ca^2+^ [21]. Researches unveiled that STIM1 was widely expressed in nonexcitable cells, including VSMCs [22]. He et al. showed that STIM1 silencing repressed cell apoptosis and reduced intracellular Ca^2+^ accumulation in cardiomyocytes [23]. Xu and his colleagues reported that resveratrol improved cardiac functional recovery via repressing STIM1-mediated regulation of intracellular Ca^2+^ [24]. Therefore, STIM1 was important for function of multiple cell types. Based on the above data, miR-641/STIM1 were hypothesized to participate in the regulatory mechanism of MIAT in AS, and whether miR-641/STIM1 pathway was responsible for the molecular mechanism by which MIAT regulated AS development needed to be explored.

The study aimed to investigate the therapeutic target for AS and reveal the role of MIAT in ox-LDL-induced VSMCs and the inner molecular mechanism.

## Methods

### Cell culture and storage

Human aorta vascular smooth muscle cells (VSMCs) were purchased from Procell (Wuhan, China) and grown in F12K medium (Procell, Wuhan, China) with 10% fetal bovine serum (FBS; Procell, Wuhan, China) and 1% penicillin/streptomycin (Procell, Wuhan, China) at 37C in an incubator with 5% CO_2_.

### Plasmid construction, oligonucleotide synthesis and cell transfection

The small interfering RNA against MIAT (si-MIAT), the mimic of miR-641 (miR-641 mimic), the inhibitor of miR-641 (miR-641 inhibitor) and controls (si-NC, miR-NC mimic and miR-NC inhibitor) were provided by GenePharma (Shanghai, China). The overexpression plasmids of MIAT (oe-MIAT) and STIM1 (pcDNA-STIM1) as well as their controls (Vector and pcDNA-NC) were built by Geneseed (Guangzhou, China). Lipofectamine 2000 (Thermo Fisher, Waltham, MA, USA) was employed for cell transfection based on the instructions of manufacturer. The sequences of oligonucleotides were si-MIAT 5-GCATTTGGTTTCAGTTCTT-3, miR-641 mimic 5-AAAGACAUAGGAUAGAGUCACCUC-3, miR-641 inhibitor 5-GAGGUGACUCUAUCCUAUGUCUUU-3, si-NC 5-GCAGTTGTTACTGTTTCTT-3, miR-NC mimic 5-UUUGUACUACACAAAAGUACUG-3 and miR-NC inhibitor 5-CAGUACUUUUGUGUAGUACAAA-3.

### 3-(4,5-Dimethylthazol-2-yl)-2,5-diphenyltetrazolium bromide (MTT) assay

VSMCs were diluted in F12K medium (Procell, Wuhan, China) and grown in 96-well plates for 16h. Then, cells were incubated with various concentrations (0, 25, 50, 75 and 100g/mL) of ox-LDL (Yeasen, Shanghai, China) for 24h or 50g/mL ox-LDL for different time (0, 12, 24, 36 and 48h). After that, MTT reagent (Solarbio, Beijing, China) was incubated with cells for 4h. Cell supernatant was discarded and dimethyl sulfoxide (Sigma, St. Louis, MO, USA) was used to dissolve formazan. The cell viability was determined by assessing the output of wavelength at 490 nm with a Varioskan LUX Multimode microplate reader (Thermo Fisher, Waltham, MA, USA).

### Quantitative real-time polymerase chain reaction (qRT-PCR)

Cultured VSMCs were collected and lysed with TransZol (TransGen, Beijing, China). RNA was isolated using an RNAsimple kit (Tiangen, Beijing, China). Then, cDNA was synthesized with a FastKing RT Kit (Tiangen, Beijing, China) or MicroRNA Reverse Transcription Kit (Thermo Fisher, Waltham, MA, USA). For determining the expression levels of MIAT, miR-641 and STIM1, SuperReal PreMix Color (Tiangen, Beijing, China) was mixed with synthesized cDNA and primers, and added into a 96-well IQ5 thermocycler (Bio-Rad, Hercules, CA, USA). After that, Data were assessed by the 2^Ct^ method. Glyceraldehyde 3-phosphate dehydrogenase (GAPDH) and U6 acted as controls. The sequences of forward and reverse primers were MIAT 5-GTGGCTCAGGAGTGCTTC-3 and 5-ACTTGCCCAGGGTTGTAG-3; miR-641 5-ACACTCCAGCTGGGGAGGTGACTCTATCCTAT-3 and 5-TGGTGTCGTGGAGTCG-3; STIM1 5-TTGGATTCTTCCCGTTCT-3 and 5-CTGGGCTGGAGTCTGTTT-3; GAPDH 5-GGTCACCAGGGCTGCTTT-3 and 5-GGAAGATGGTGATGGGATT-3; U6 5-CTCGCTTCGGCAGCACA-3 and 5-AACGCTTCACGAATTTGCGT-3.

### Cell colony formation assay

VSMCs were seeded in 6-well plates for 16h and treated with 50g/mL ox-LDL (Yeasen, Shanghai, China). Twenty-four hours later, si-MIAT, miR-641 inhibitor, miR-641 mimic or pcDNA-STIM1 was transfected into the cells at ~70% confluence with controls according to the defined purposes. Then, cells were cultured for 2 weeks. F12K medium (Procell, Wuhan, China) was renewed every 3 days during culture. The forming colonies were immobilized with paraformaldehyde (Sigma, St. Louis, MO, USA) and then dyed with crystal violet (Sigma, St. Louis, MO, USA). Cell colony-forming ability was determined by assessing the number of colonies. A colony was deemed when cell numbers over 50.

### DNA content quantitation assay

Cell cycle was detected by DNA content quantitation assay. In short, cultured VSMCs were collected and fixed with cold ethanol (Millipore, Bradford, MA, USA). Cells were precipitated by centrifugation at 300g for 5min, and incubated with RNase A (Solarbio, Beijing, China) at 37C for 30min. After that, propidium iodide (PI; Solarbio, Beijing, China) was employed to stain cells at 4C for 30min. Samples were assessed by a flow cytometry (Thermo Fisher, Waltham, MA, USA).

### Wound-healing assay

VSMCs were grown in 6-well plates and treated with different purposes. Cells were cultured until its confluence reached about 100%. Cell wounds were created with 10-L pipette tips and cells were then cultivated in FBS-free F12K medium (Procell, Wuhan, China). At 24h after culture, the width of the wounds was measured under an inverted microscope (Nikon, Tokyo, Japan) with a 40() magnification, and cell migratory ability was determined by analyzing wound width.

### Western blot analysis

Cells were harvested and lysed with RIPA buffer (Sigma, St. Louis, MO, USA) possessing proteinase K (Millipore, Bradford, MA, USA). Then, proteins were denaturalized at 95C, and lysates were loaded onto 12% bis-tris-acrylamide gel (Thermo Fisher, Waltham, MA, USA) to separate proteins. The protein bands were transferred onto polyvinylidene fluoride membranes (Millipore, Bradford, MA, USA), which were then immersed in 5% non-fat milk (Solarbio, Beijing, China). Subsequently, the membranes were incubated with anti-proliferating cell nuclear antigen (anti-PCNA) (1:1500; Affinity, Nanjing, China), anti-nuclear proliferation marker (anti-Ki-67) (1:1500; Affinity, Nanjing, China), anti-phospho-focal adhesion kinase (anti-p-FAK) (1:1000; Affinity, Nanjing, China), anti-Ago2 (1:1000; Affinity, Nanjing, China), anti-IgG (1:2000; Abcam, Cambridge, UK), anti-STIM1 (1:1500; Affinity, Nanjing, China) and anti-GAPDH (1:15,000, Affinity, Nanjing, China). The membranes were incubated with horseradish peroxidase-marked secondary antibody (1:5000; Affinity, Nanjing, China). The protein bands were presented with RapidStep ECL Reagent (Millipore, Bradford, MA, USA), and protein expression was determined by Image J software (NIH, Bethesda, MD, USA).

### Transwell migration and invasion assays

The migrated and invaded cells were determined by transwell chambers without or with Matrigel (Corning, Madison, New York, USA). In brief, cells were seeded in the upper chambers containing FBS-free F12K medium (Procell, Wuhan, China) after treated with ox-LDL, si-MIAT, si-NC, miR-641 inhibitor, miR-NC inhibitor, miR-641 mimic, miR-NC mimics, pcDNA-STIM1 or pcDNA-NC. In the lower chambers, F12K medium containing 15% FBS (Procell, Wuhan, China) was added. Twenty-four hours later, cell supernatant was discarded, and cells were incubated with paraformaldehyde (Sigma, St. Louis, MO, USA) and crystal violet (Sigma, St. Louis, MO, USA), respectively. Results were determined via counting the number of cells in the lower chambers under a microscope (Nikon, Tokyo, Japan) at a 100() magnification.

### Dual-luciferase reporter assay

The binding sites between miR-641 and MIAT or STIM1 were firstly assessed through starbase online database (http://starbase.sysu.edu.cn/agoClipRNA.php?source=mRNA). And the wild-type (WT) and mutant (MUT) plasmids of MIAT and the 3-untranslated region (3UTR) of STIM1 were built by Geneseed Co., Ltd. (Guangzhou, China), and named as WT-MIAT, WT-STIM1 3UTR, MUT-MIAT and MUT-STIM1 3UTR. Constructed plasmids and synthesized oligonucleotides were transfected into the VSMCs at ~70% confluence using Lipofectamine 2000 (Thermo Fisher, Waltham, MA, USA). Post-culture of 48h, the cells were collected and lysed using lysis buffer (Promega, Madison, WI, USA). Luciferase activities were detected with a dual luciferase reporter assay kit (Promega; Madison, WI, USA). *Renilla* luciferase activity served as a control.

### RNA immunoprecipitation (RIP) assay

MiR-641 mimic was transfected into VSMCs with miR-NC mimics as a control. Post-transfection of 48h, the cells were collected and lysed with RIP lysis buffer (Millipore, Bradford, MA, USA) containing protease inhibitor (Millipore, Bradford, MA, USA). After that, lysates were incubated with the magnetic beads bound with anti-Ago2 (RIP-Ago2; Abcam, Cambridge, UK) or anti-IgG (RIP-IgG; Abcam, Cambridge, UK). Twenty-four hours later, the magnetic beads were washed, and MIAT and STIM1 expression were determined by qRT-PCR.

### Statistical analysis

Data derived from three independent duplicate tests were assessed by SPSS 21.0 software (IBM, Somers, NY, USA). Results were expressed as meansstandard deviations (SD). Significant differences were compared with two-tailed Students *t*-tests between the two groups or one-way analysis of variance (ANOVA) with Tukeys test among three or more groups. Statistical significance was defined when *P* value<0.05.

## Results

### MIAT expression was upregulated in ox-LDL-induced VSMCs

**Fig. 1:**
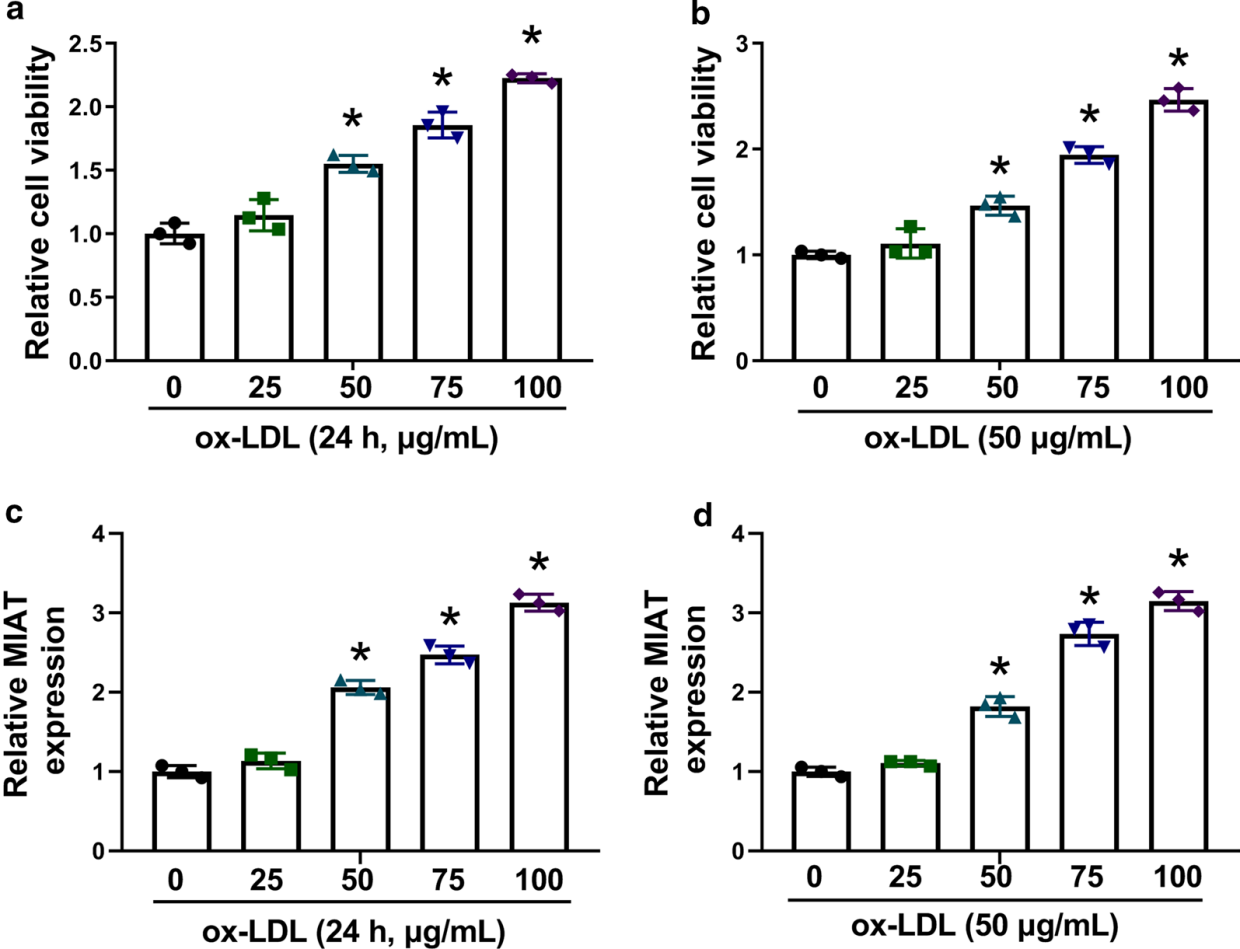
MIAT expression was increased in ox-LDL-incubated VSMCs. **a** MTT assay was employed to demonstrate the impacts of various concentrations of ox-LDL (0, 25, 50, 75 and 100g/mL) on the viability of VSMCs at 24h after incubation. **b** The effects of 50g/mL ox-LDL on the viability of VSMCs were demonstrated by MTT assay at various time points (0, 12, 24, 36 and 48h) after incubation. **c** The impacts of various doses of ox-LDL (0, 25, 50, 75 and 100g/mL) on MIAT expression at 24h after incubation were revealed by qRT-PCR. **d** The effects of 50g/mL ox-LDL on MIAT expression were determined by qRT-PCR at various time points (0, 12, 24, 36 and 48h) after incubation. *P<0.05

In order to determine the impact of ox-LDL treatment on MIAT expression, the reasonable induction dose and induction time of ox-LDL for VSMCs were firstly determined. Result presented ox-LDL exposure (50, 75 and 100g/mL) promoted cell viability in a dose-dependent manner (Fig.1a). Additionally, data showed that 50g/mL ox-LDL also facilitated cell viability in a time-dependent manner with a minimum incubation time of 24h (Fig.1b). Based on these data, VSMCs were incubated with 50g/mL ox-LDL for 24h in further ox-LDL-related study. The impacts of various concentrations of ox-LDL (0, 25, 50, 75 and 100g/mL) on MIAT expression were continued to be explored. Our data displayed MIAT expression was substantially upregulated in VSMCs treated by ox-LDL (50, 75 and 100g/mL) in a concentration-dependent manner (Fig.1c). Meanwhile, results exhibited 50g/mL ox-LDL increased MIAT expression after culture for 24h (Fig.1d). These data demonstrated that MIAT might be involved in ox-LDL-induced VSMC cell disorder.

### MIAT silencing attenuated ox-LDL-induced cell proliferation, migration and invasion in VSMCs

**Fig. 2:**
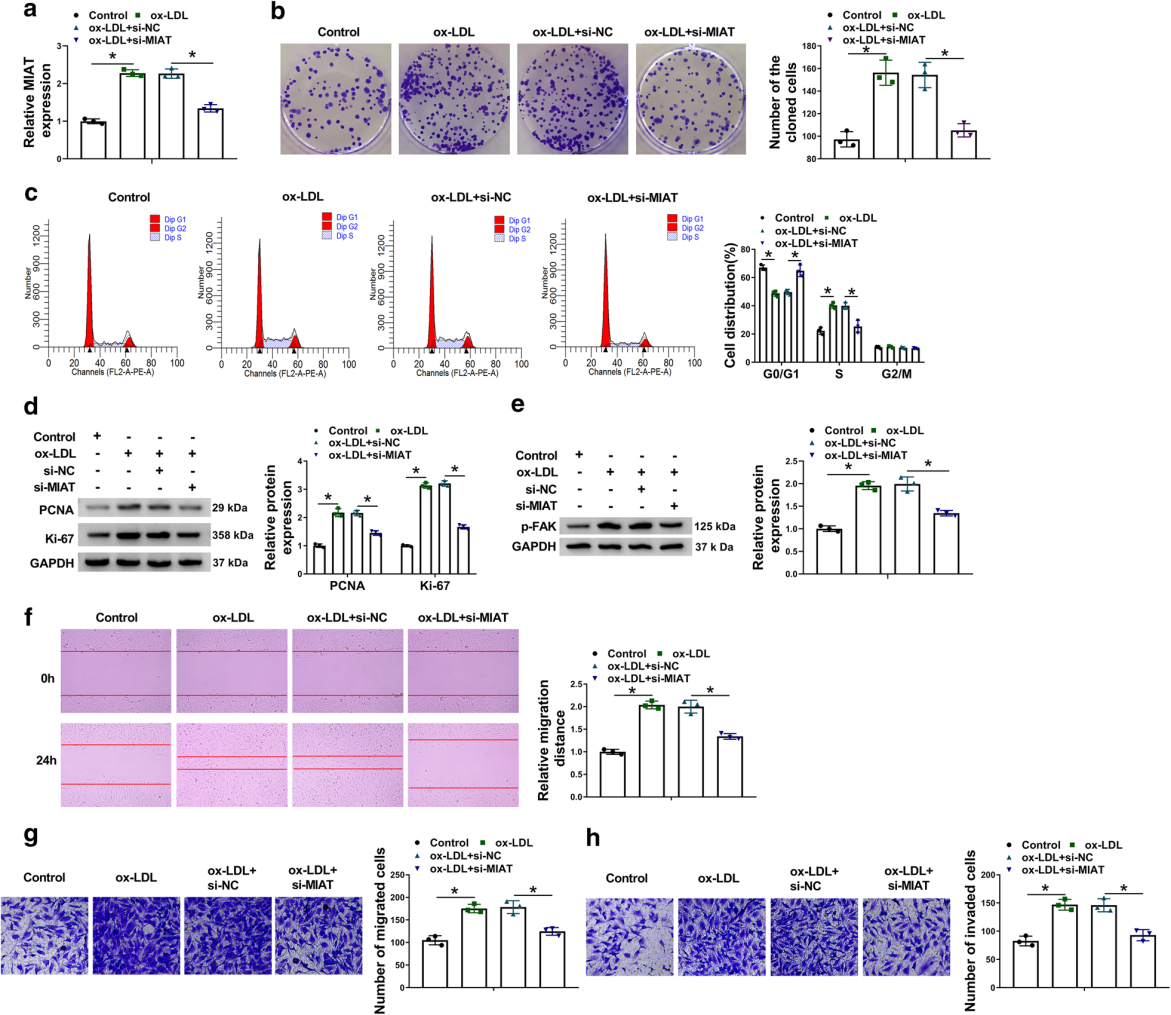
MIAT regulated ox-LDL-mediated VSMC processes. **a** The impacts between ox-LDL treatment and MIAT silencing on MIAT expression were revealed by qRT-PCR in VSMCs. **b**, **c** The effects between ox-LDL treatment and MIAT silencing on the proliferation of VSMCs were determined by cell colony formation and DNA content quantitation assays. **d**, **e** Western blot analysis was conducted to determine the impacts between ox-LDL treatment and MIAT absence on the protein expression of PCNA, Ki-67 and p-FAK. **f**, **g** Wound-healing and transwell migration assays were employed to explain the impacts between ox-LDL treatment and MIAT knockdown on the migration of VSMCs. **h** The impacts between ox-LDL treatment and MIAT knockdown on the invasion of VSMCs were demonstrated by transwell invasion assay. *P<0.05

To further determine whether MIAT participated in the regulation of ox-LDL-induced cell proliferation, migration and invasion, si-MIAT was transfected into ox-LDL-induced VSMCs with control groups. Results firstly showed that MIAT knockdown impaired the promoting impact of ox-LDL on MIAT expression (Fig.2a). Cell colony formation assay showed that ox-LDL treatment enhanced the colony-forming ability of VSMCs, whereas this impact was restored after MIAT silencing (Fig.2b). Meanwhile, data presented that ox-LDL exposure promoted cell arrest in S phase, while this impact was hindered after downregulation of MIAT (Fig.2c). To further explain the effect of MIAT silencing on ox-LDL-triggered cell proliferation, we detected the protein expression of proliferation-related PCNA and Ki-67. As shown in Fig.2d, ox-LDL treatment upregulated the protein expression of PCNA and Ki-67; however, MIAT knockdown attenuated these effects. The above data suggested that MIAT silencing restored ox-LDL-triggered cell proliferation. Subsequently, western blot analysis presented that the protein expression of metastasis-related p-FAK was increased after treatment of ox-LDL, while the effect was impaired after transfection of si-MIAT (Fig.2e). Also, the enhanced migratory and invasive capacities of VSMCs by ox-LDL were reversed after MIAT absence (Fig.2fh). The results from Fig.2eh indicated that MIAT silencing restored ox-LDL-triggered cell migration and invasion. Taken together, the above evidences demonstrated MIAT knockdown assuaged ox-LDL-induced cell disorders.

### MiR-641 was directly associated with MIAT and STIM1 in VSMCs

**Fig. 3:**
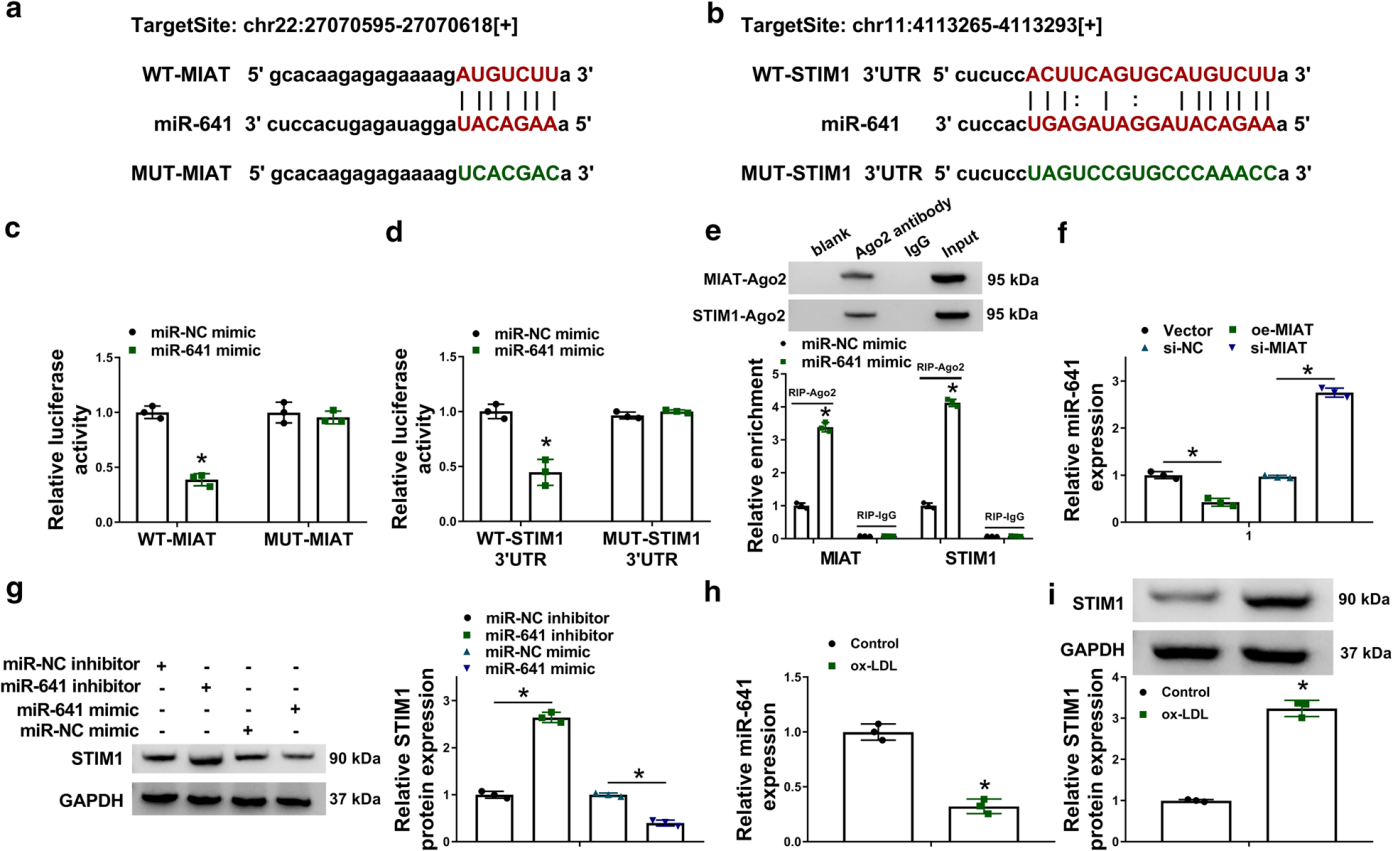
MiR-641 directly bound to MIAT and STIM1 in VSMCs. **a**, **b** The putative binding sites of miR-641 for both MIAT and STIM1 were predicted by starbase online database. **ce**The targeting relationship between miR-641 and MIAT or STIM1 was proved by dual-luciferase reporter and RIP assays. **f** The impacts of MIAT overexpression and silencing on miR-641 expression were revealed by qRT-PCR in VSMCs. **g** The impacts of miR-641 inhibitor and mimic on STIM1 protein expression were determined by qRT-PCR. **h** MiR-641 and STIM1 expression were detected by qRT-PCR or western blot in ox-LDL-induced VSMCs and control group. *P<0.05

**Fig. 4:**
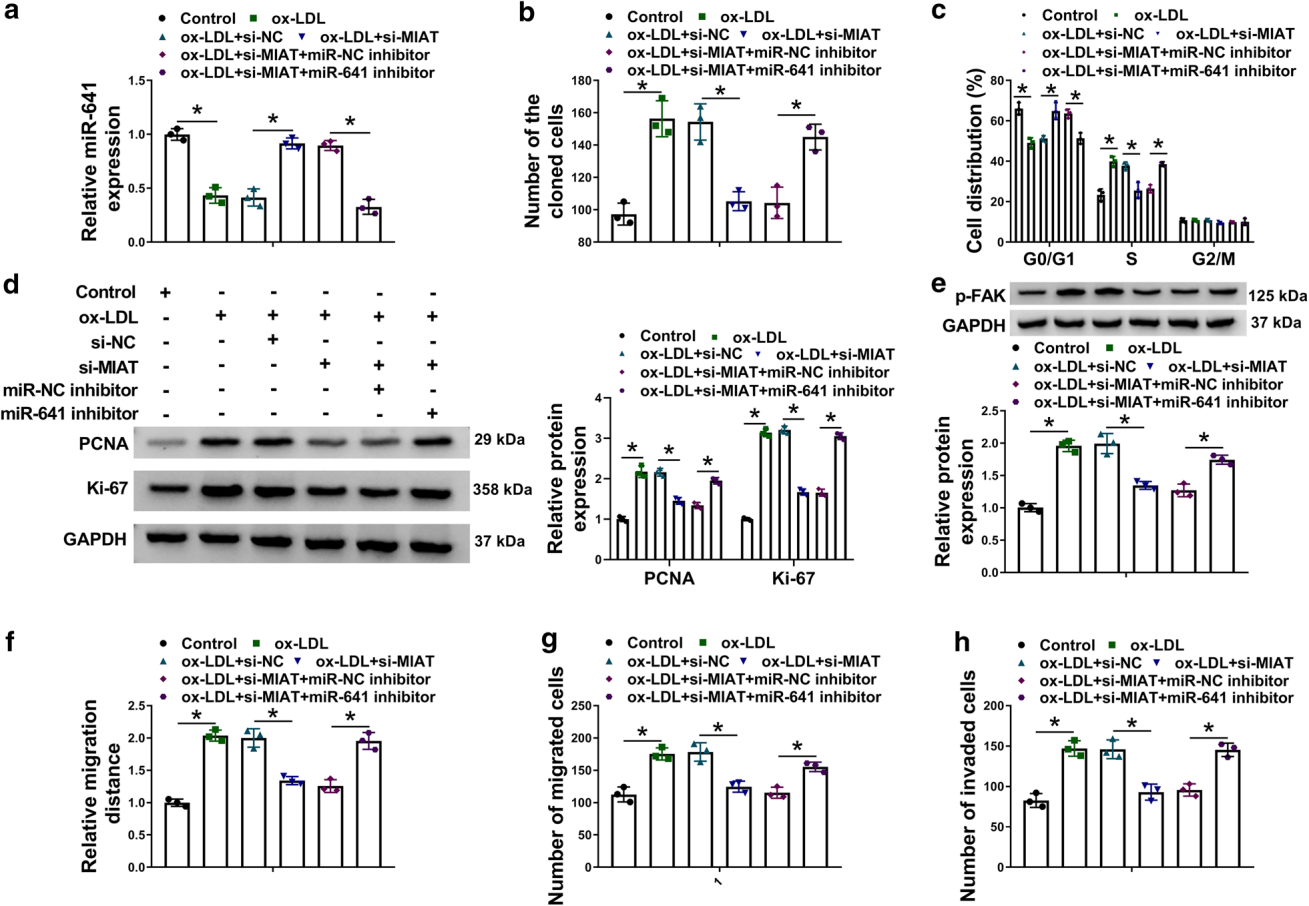
MIAT silencing hindered ox-LDL-induced cell proliferation, migration and invasion by associating with miR-641.**a** The effects between MIAT silencing and miR-641 inhibitor on miR-641 expression were revealed by qRT-PCR in ox-LDL-induced VSMCs. **b**, **c** The impacts between MIAT absence and miR-641 inhibitor on cell proliferation were determined by cell colony formation and DNA content quantitation assays in ox-LDL-induced VSMCs. **de**The impacts between MIAT knockdown and miR-641 inhibitor on the protein expression of PCNA, Ki-67 and p-FAK were investigated by western blot analysis in ox-LDL-induced VSMCs. **f**, **g**Wound-healing and transwell migration assays were employed to disclose the impacts between MIAT silencing and miR-641 inhibitor on cell migration in ox-LDL-induced VSMCs. **g** Transwell invasion assay was performed to reveal the influences between MIAT knockdown and miR-641 inhibitor on VSMC invasion under ox-LDL treatment. *P<0.05

The study continued to explore the underlying mechanism of MIAT in regulating ox-LDL-mediated VSMC process. According to the results predicted by starbase online database, we found that MIAT contained the binding sites of miR-641, which was further found to have complementary sites of STIM1 3UTR (Fig.3a, b). To further determine whether both MIAT and STIM1 were associated with miR-641, dual-luciferase reporter and RIP assays were employed. Before that, we detected the overexpression or knockdown efficiency of miR-641 mimic, miR-641 inhibitor, oe-MIAT and si-MIAT, and results were shown in Figure S1A-C. Then, as shown in Fig.3c, d, the relative luciferase activity was significantly repressed in miR-641 mimic and WT-MIAT co-transfection group as well as in miR-641 mimic and WT-STIM1 3UTR co-transfection group, whereas the relative luciferase activity had no obvious change in miR-641 mimic and MUT-MIAT co-transfection group or in miR-641 mimic and MUT-STIM1 3UTR co-transfection group. Meanwhile, RIP assay presented both MIAT and STIM1 pulled down by RIP-Ago2 was significantly enriched in the VSMCs transfected with miR-641 mimic as compared with control groups (Fig.3e). Additionally, it was found that miR-641 expression was significantly decreased after overexpression of MIAT, but upregulated by MIAT silencing (Fig.3f). And qRT-PCR data showed that miR-641 inhibitor substantially upregulated STIM1 protein expression, but miR-641 mimic downregulated STIM1 protein expression (Fig.4g). The above results explained that miR-641 was directly associated with MIAT and STIM1 in VSMCs. Furthermore, data presented miR-641 expression was notably decreased and STIM1 protein expression was increased in ox-LDL-induced VSMCs (Fig.3h, i), suggesting that miR-641 and STIM1 might participate in ox-LDL-induced VSMC disorders (Additional file 1).

### MIAT silencing repressed ox-LDL-induced cell proliferation, migration and invasion by binding to miR-641 in VSMCs

**Fig. 5:**
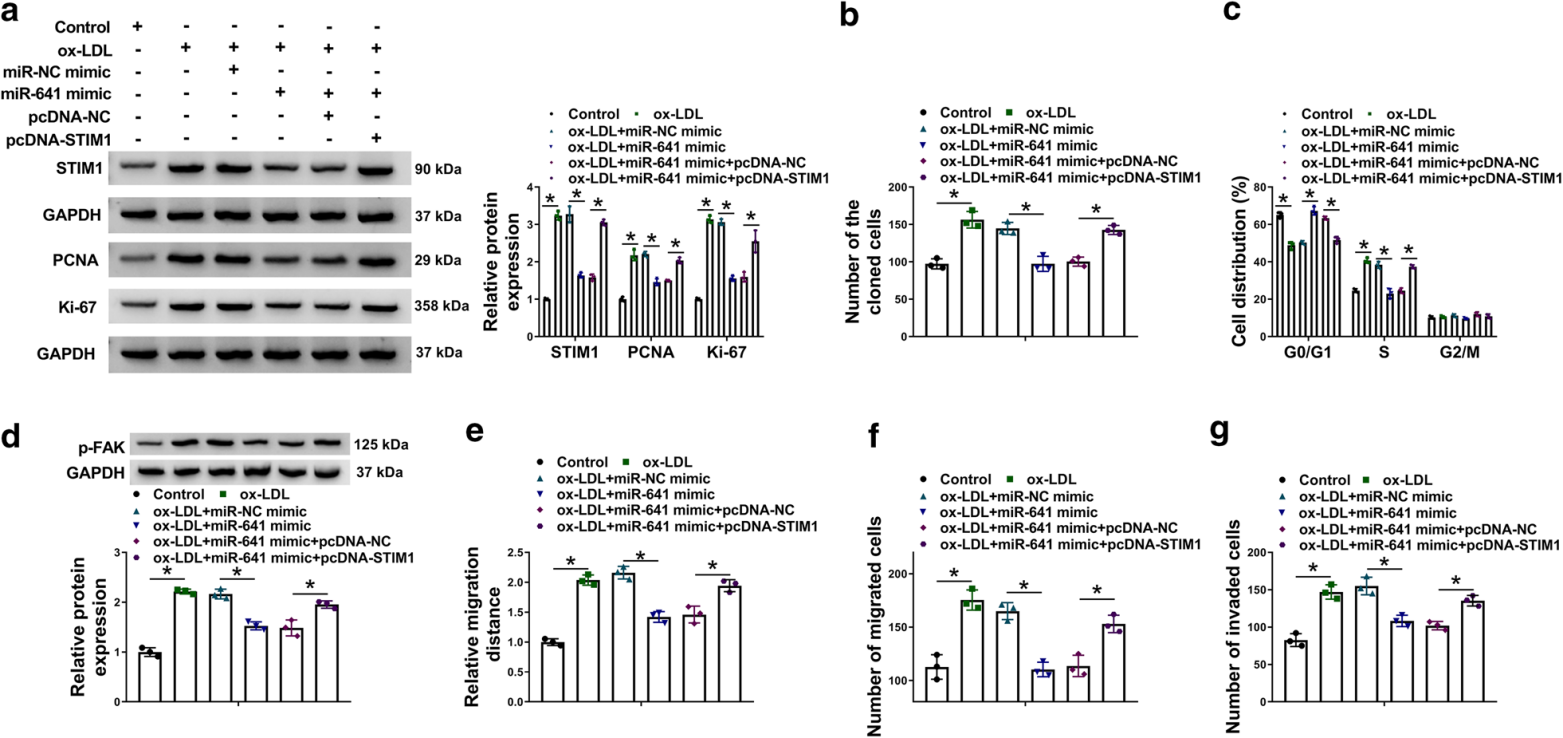
MiR-641 hindered ox-LDL-induced cell proliferation, migration and invasion via interacting with STIM1.**a**, **d**The impacts between miR-641 and STIM1 overexpression on STIM1, PCNA, Ki-67 and p-FAK protein expression under ox-LDL treatment were detected by western blot analysis in VSMCs. **b**, **c**The impacts between miR-641 mimic and STIM1 overexpression on cell proliferation under ox-LDL treatment were revealed by cell colony formation and DNA content quantitation assays in VSMCs. **e**, **f**The impacts between miR-641 and STIM1 overexpression on cell migration under ox-LDL treatment were investigated by wound-healing and transwell migration assays in VSMCs. **g** The effects between miR-641 mimic and STIM1 overexpression on cell invasion under ox-LDL treatment were revealed by transwell invasion assay in VSMCs. *P<0.05

Whether MIAT regulated ox-LDL-induced VSMC process by interacting with miR-641 was revealed in this part. Results initially exhibited MIAT silencing attenuated the repressive impact of ox-LDL exposure on miR-641 expression, whereas this impact was reversed after transfection of miR-641 inhibitor (Fig.5a). Additionally, si-MIAT-mediated repressive impact on cell colony-forming ability was restored by miR-641 inhibitor in ox-LDL-induced VSMCs (Fig.5b). DNA content quantitation assay also presented MIAT silencing promoted cell arrest in G0/G1 phase in ox-LDL-induced VSMCs, which was hindered after transfection of miR-641 inhibitor (Fig.5c). Meanwhile, western blot analysis showed the protein expression of PCNA, Ki-67 and p-FAK was significantly downregulated after silencing of MIAT in ox-LDL-induced VSMCs, but these effects were hindered by miR-641 inhibitor (Fig.5de). Furthermore, MIAT silencing repressed cell migration and invasion in ox-LDL-induced VSMCs; however, these results were attenuated by miR-641 inhibitor (Fig.5fh). All in all, these data manifested that MIAT modulated ox-LDL-induced cell proliferation, migration and invasion by sponging miR-641 in VSMCs.

### MiR-641 repressed ox-LDL-induced cell proliferation, migration and invasion by targeting STIM1 in VSMCs

Given the associated relationship between miR-641 and STIM1, whether miR-641 modulated ox-LDL-induced cell proliferation, migration and invasion by binding to STIM1 was further explored. Results firstly showed that miR-641 mimic attenuated the upregulating impact of ox-LDL treatment on STIM1 protein expression, whereas ectopic STIM1 expression hindered this impact (Fig.4a). Additionally, the impacts of ox-LDL on cell colony-forming ability and S phase cell arrest were impaired by miR-641 mimic, which was reversed after transfection of pcDNA-STIM1 (Fig.4b, c). As expected, miR-641 mimic restored ox-LDL-induced promotion on the PCNA and Ki-67 protein levels, but STIM1 overexpression hindered these influences (Fig.4a). Subsequently, miR-641 mimic-mediated downregulation of p-FAK protein expression under ox-LDL treatment was attenuated by ectopic STIM1 expression (Fig.4d). Also, we found the enhanced migratory and invasive capacities of VSMCs by ox-LDL were restrained after miR-641 mimic transfection; however, enforced expression of STIM1 abolished these impacts (Fig.4eg). Collectively, these findings explained that miR-641 repressed ox-LDL-induced cell proliferation, migration and invasion via interacting with STIM1.

### MIAT regulated STIM1 expression by associating with miR-641

**Fig. 6:**
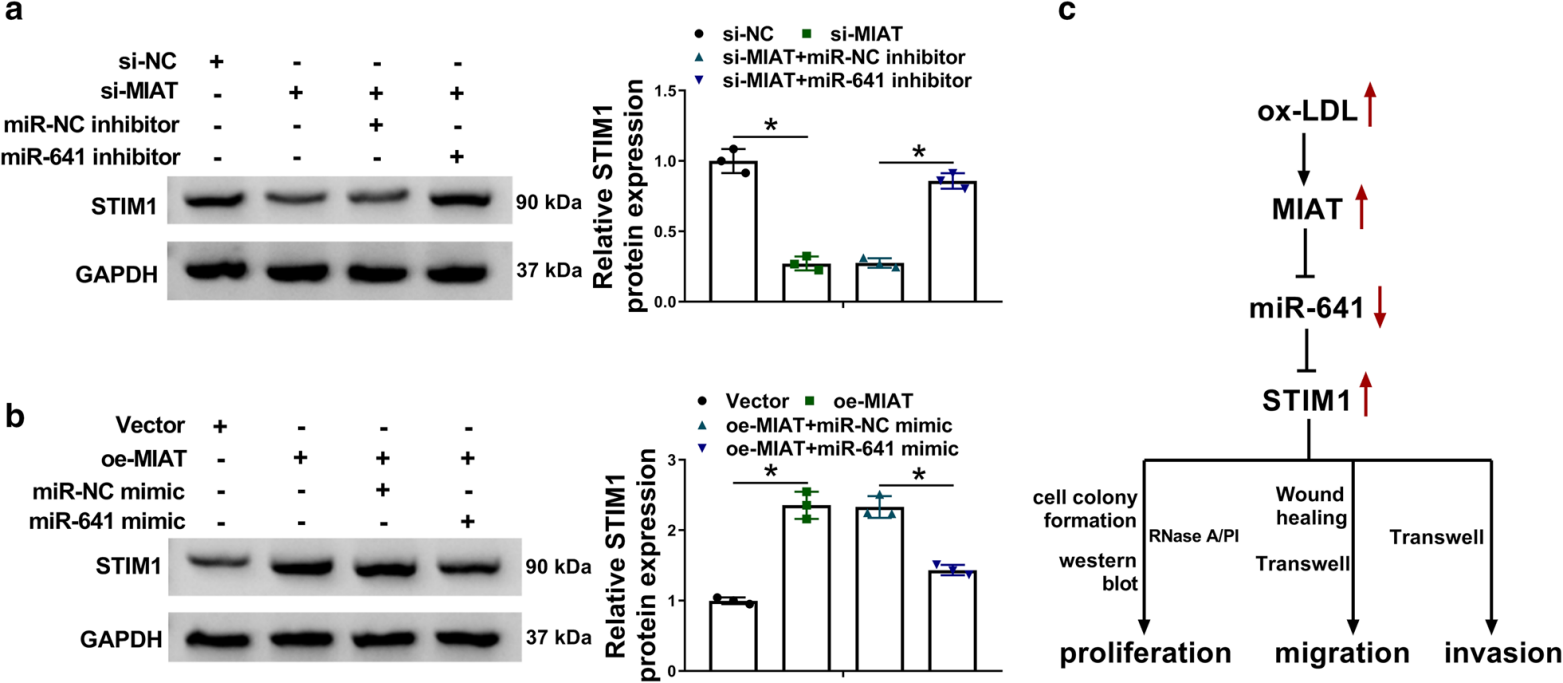
MIAT modulated STIM1 expression by interacting with miR-641. **a** The impacts between MIAT silencing and miR-641 inhibitor on STIM1 protein expression were determined by western blot analysis in VSMCs. **b** The impacts between MIAT overexpression and miR-641 mimic on STIM1 protein expression were determined by western blot analysis in VSMCs. **c** The schematic diagram of the mechanism underlying MIAT regulating ox-LDL-induced VSMC disorders. *P<0.05

Whether MIAT controlled STIM1 expression by associating with miR-641 was continued to be revealed. To this end, si-MIAT and miR-641 inhibitor were co-transfected into VSMCs with control groups. Result showed that MIAT silencing significantly downregulated STIM1 protein expression, whereas this effect was restored after transfection of miR-641 inhibitor (Fig.6a). On the contrary, MIAT overexpression increased STIM1 protein expression, but this effect was restrained by miR-641 mimic (Fig.6b). These results demonstrated that MIAT could modulate STIM1 expression by binding to miR-641. Thus, we came a conclusion that ox-LDL treatment upregulated MIAT expression, which further sponged miR-641 to induce STIM1, thereby promoting cell proliferation, migration and invasion (Fig.6c).

## Discussion

Although many achievements have been created in revealing the pathogenesis of AS, AS still poses a heavy threat to human health [25]. Previous researches showed that lots of lncRNAs participated in regulating the process of cardiovascular diseases, including AS [26, 27]. As reported, lnc00113 could accelerate cell proliferation and migration via mediating phosphoinositide 3-kinase (PI3K)/protein kinase B (Akt)/mTOR pathway in AS [28]. Xia et al. indicated lncRNA gm4418 accelerated cell apoptosis and deteriorated hypertensive cerebral arteriosclerosis [29]. In another example, retinal non-coding RNA3 (RNCR3) knockdown aggravated hypercholesterolemia and repressed the proliferative and migratory abilities of VSMCs [30]. Herein, we found MIAT silencing repressed cell proliferation, migration and invasion by regulating miR-641/STIM1 axis in ox-LDL-induced VSMCs.

Previous studies presented that MIAT participated in the regulation of AS progression. For example, MIAT promoted atherosclerotic plaques formation, angiogenesis and inflammation, and upregulated lipid content [11, 13]. Zhong et al. explained MIAT accelerated cell proliferation, but restrained cell apoptosis via sponging miR-181b [31]. Additionally, it was found that MIAT was augmented and enhanced cell proliferative and metastatic capacities in ox-LDL-stimulated VSMCs [14, 32]. In this paper, we also found MIAT was elevated in ox-LDL-induced VSMCs. MIAT absence restored ox-LDL-induced proliferation, migration and invasion in VSMCs. As reported, PCNA takes part in many aspects of DNA replication and is regarded as an important regulator of vital events at the replication fork [33]; Ki-67 has a conserved leucine/arginine rich C-terminusand that can bind to DNA, thereby promoting chromatin compaction [34]; FAK is a tyrosine kinase and can be recruited to sites of integrin clustering or focal adhesions, and its phosphorylation status is revealed to be correlated with cell metastasis [35]. In the study, we found MIAT absence also attenuated the promoting effects of ox-LDL on the protein expression of PCNA, Ki-67 and p-FAK. All these evidences suggested the repressing role of MIAT in ox-LDL-induced VSMC disorders. Considering that lncRNA-miRNA-mRNA network played a vital part in unveiling the pathogenesis of cardiovascular diseases [36], the miRNA and mRNA associated with MIAT were sought. Results exhibited that MIAT interacted with miR-641, which was further revealed to target STIM1.

Current researches revealed that miR-641 was closely correlated with disease progression. Researches indicated that miR-641 repressed cell proliferation in lung cancer [37] and gastric cancer [38]. Zhang et al. explained that miR-641 participated in osteoarthritis process by regulating extracellular matrix metabolism and inflammation [39]. Additionally, miR-641 was reported to repress cisplatin resistance and erlotinib sensitivity in lung cancer [40, 41]. Here, the paper was the first one to report the role of miR-641 in AS process. We found miR-641 expression was decreased in ox-LDL-treated VSMCs, and miR-641 inhibitor restrained MIAT knockdown-mediated impacts, which suggested that miR-641 served as a suppressor in AS evolution. Meanwhile, the evidences from our study suggested that MIAT regulated ox-LDL-induced development of VSMCs via interacting with miR-641.

Ca^2+^ influx into cells is commonly mediated by capacitative Ca^2+^ entry pathway, which is reported to be modulated by STIM (STIM1 and STIM2) as well as Orai proteins [42]. This finding implied that STIM1 played a crucial part in biological behaviors of various cells. Coincidently, we found STIM1 was a target gene of miR-641. Herein, STIM1 was overexpressed in ox-LDL-treated VSMCs. And ectopic STIM1 expression impaired miR-641-mdiated effects on cell proliferation and metastasis in ox-LDL-stimulated VSMCs. The evidences from our research implicated STIM1 served as a promoter in the progression of ox-LDL-stimulated VSMCs, which was proved by the existed evidences [43, 44]. In the meantime, the above data implied that miR-641 repressed the progression of VSMCs stimulated by ox-LDL via binding to STIM1. Given the associated relationships between MIAT and miR-641 as well as between miR-641 and STIM1, whether MIAT modulated STIM1 by binding to miR-641 was continued to be illustrated. Rescue experiments showed MIAT silencing significantly decreased STIM1 protein expression, whereas miR-641 inhibitor attenuated this impact. Additionally, miR-641 mimic also restored the upregulating impact of MIAT overexpression on STIM1 protein expression. These evidences suggested MIAT could control STIM1 expression through associating with miR-641.

However, there are some limitations that should be considered when interpreting our findings. First, the study only focuses on the roles of MIAT in regulating ox-LDL-induced cell injury in vitro, and the in vivo data are lacking in the present research, which is expected to be explored in future. To address the question, we would study the biological role of MIAT using Apoe/ mouse. Additionally, ox-LDL-induced VSMC injury might not only attribute to MIAT/miR-641/STIM1 pathway, and there might were other singling pathways, which needed to be explored by more assays and bioinformatics methods.

## Conclusions

Collectively, MIAT knockdown hindered cell proliferation, migration and invasion in ox-LDL-treated VSMCs. MiR-641 inhibitor reversed MIAT silencing-mediated action under ox-LDL treatment. Additionally, MIAT was associated with miR-641, which was further proved to target STIM1. STIM1 overexpression also attenuated miR-641-mediated effects on the process of VSMCs stimulated by ox-LDL. All in all, MIAT modulated ox-LDL-induced cell proliferation, migration and invasion via miR-641/STIM1 pathway in VSMCs. Our finding not only provides a new mechanism for unveiling the pathogenesis of AS, but also lays a foundation for studying lncRNA-directed AS therapy.

## Supplementary Information

**Additional file 1.** **Figure S1**: The expression of miR-641 and MIAT was detected by qRT-PCR. **a** The expression of miR-641 was determined by qRT-PCR in the VSMCs transfected with miR-NC mimic or miR-641 mimic. **b** QRT-PCR was employed to detect miR-641 expression in the VSMCs transfected with miR-NC inhibitor or miR-641 inhibitor. **c** The effects of oe-MIAT and si-MIAT on MIAT expression were checked by qRT-PCR in VSMCs

## Data Availability

All data generated and/or analyzed during the current study are available from the corresponding author on reasonable request.

## Abbreviations

AS: Atherosclerosis
MIAT: myocardial infarction associated transcript
VSMCs: vascular smooth muscle cells
PCNA: proliferating cell nuclear antigen
