# Supplementary figures and images for: Long non-coding RNA MIAT regulates ox-LDL-induced cell proliferation, migration and invasion by miR-641/STIM1 axis in human vascular smooth muscle cells
Source: BMC Cardiovasc Disord. 2021 May 20;21:248. doi: 10.1186/s12872-021-02048-9 (PMC8139145; doi:10.1186/s12872-021-02048-9)

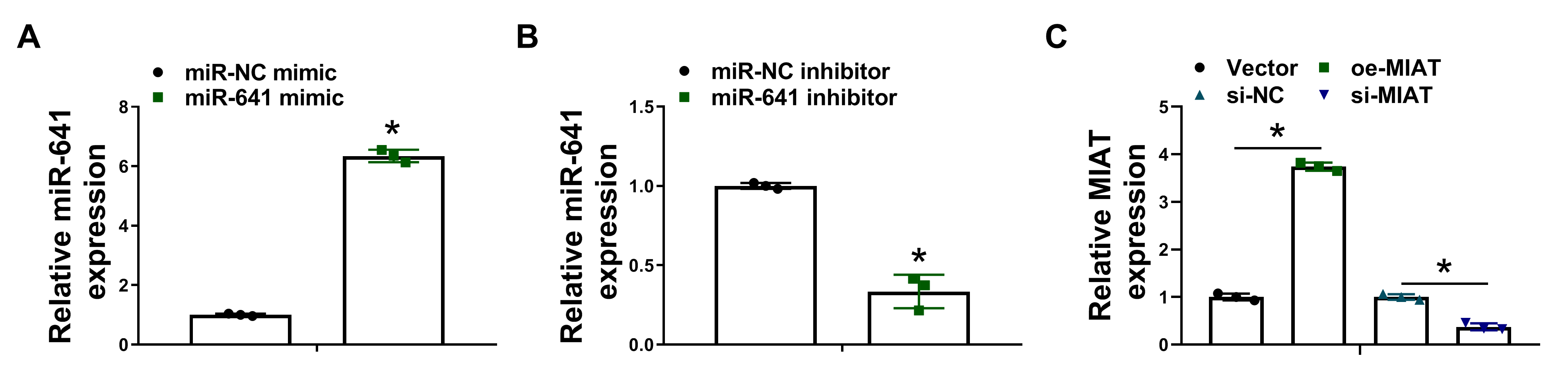

Supplement: Supplementary file 1 — Additional file 1. Figure S1: The expression of miR-641 and MIAT was detected by qRT-PCR. a The expression of miR-641 was determined by qRT-PCR in the VSMCs transfected with miR-NC mimic or miR-641 mimic. b QRT-PCR was employed to detect miR-641 expression in the VSMCs transfected with miR-NC inhibitor or miR-641 inhibitor. c The effects of oe-MIAT and si-MIAT on MIAT expression were checked by qRT-PCR in VSMCs [file 12872_2021_2048_MOESM1_ESM.tif]
